# Conformationally locked 7-aryl tetrahydroisoquinolines as dual acetylcholinesterase inhibitors and antioxidants: role of intramolecular hydrogen bonding and aryl electronics

**DOI:** 10.3389/fphar.2026.1797738

**Published:** 2026-04-08

**Authors:** Valentina Ríos, Camila Linares-Pipón, Cristián Maulén, Alejandro Castro-Álvarez, Ben Bradshaw, Javier Romero-Parra, Mauricio A. Cuellar, Maximiliano Martínez-Cifuentes, Claudio Parra

**Affiliations:** 1 Departamento de Química Orgánica, Facultad de Ciencias Químicas, Universidad de Concepción, Concepción, Chile; 2 Departamento de Ciencias Preclínicas, Facultad de Medicina, Universidad de La Frontera, Temuco, Chile; 3 Laboratori de Química Orgànica, Facultat de Farmacia, IBUB, Universitat de Barcelona, Barcelona, Spain; 4 Departamento de Química Orgánica y Fisicoquímica, Facultad de Ciencias Químicas y Farmacéuticas, Universidad de Chile, Santiago, Chile; 5 Centro de Investigación Farmacopea Chilena, Escuela de Química y Farmacia, Facultad de Farmacia, Universidad de Valparaíso, Valparaíso, Chile

**Keywords:** antioxidants, density functional theory, molecular docking, multitarget-directed ligands, structure–activity relationships, tetrahydroisoquinolin

## Abstract

**Background:**

Alzheimer’s disease (AD) is a multifactorial neurodegenerative disorder in which cholinergic dysfunction and oxidative stress act as synergistic contributors to cognitive decline and neuronal damage. Multitarget-directed ligands (MTDLs) capable of modulating both acetylcholinesterase (AChE) activity and oxidative stress pathways are promising candidates for disease-modifying therapies.

**Methods:**

A series of conformationally locked 7-aryl tetrahydroisoquinoline derivatives was synthesized via a tandem cross-metathesis/Michael/annulation strategy. These compounds feature a stable intramolecular O–H···O=C hydrogen bond that enforces a quasi-planar geometry and modulates the electronic properties of the aryl substituent. The compounds were evaluated for their AChE inhibitory activity using Ellman’s assay and for antioxidant capacity using the oxygen radical absorbance capacity (ORAC) assay. Molecular docking studies were performed to analyze binding modes within AChE’s aromatic gorge, while density functional theory (DFT) calculations were conducted to assess the thermodynamics of hydrogen atom transfer (HAT) and ionization potential (IP) related to antioxidant behavior.

**Results:**

All derivatives exhibited micromolar AChE inhibition (IC_50_ = 33–54 µM), with structure–activity relationships driven by the electronic nature and π-polarizability of the 7-aryl ring. Docking results revealed a conserved, π-dominated binding pose within the enzyme’s active site. ORAC measurements showed substituent-dependent radical-scavenging activity consistent with the electronic trends observed in enzyme inhibition. DFT calculations indicated a thermodynamic preference for hydrogen atom transfer (HAT) from benzylic C–H over phenolic O–H cleavage, supporting the observed antioxidant profile.

**Conclusion:**

This study identifies 7-aryl tetrahydroisoquinolines as a novel, mechanistically coherent scaffold for the development of dual-acting neuroprotective agents targeting both cholinergic dysfunction and oxidative stress in Alzheimer’s disease. Their tunable electronic properties and preorganized geometry offer a promising foundation for further optimization within the multitarget therapeutic framework.

## 1 Introduction

Alzheimer’s disease (AD) is a progressive, irreversible, and multifactorial neurodegenerative disorder clinically characterized by memory impairment, cognitive decline, and the eventual loss of functional autonomy (Dubey et al., 2018). Together with other forms of dementia, AD constitutes a major and escalating global health burden, affecting millions of individuals worldwide, with both incidence and prevalence expected to rise sharply as populations continue to age (Yu et al., 2025; Fu et al., 2025; Monfared et al., 2024). Extensive epidemiological and mechanistic studies have revealed that AD arises from a convergence of pathological processes, including amyloid-β (Aβ) accumulation, deterioration of cholinergic neurotransmission, dysregulated calcium homeostasis, τ-protein hyperphosphorylation, mitochondrial dysfunction, and chronic oxidative stress (Zhang et al., 2024). Neuropathologically, AD is defined by selective degeneration of cholinergic neurons, the presence of intracellular neurofibrillary tangles composed of hyperphosphorylated τ, and extracellular senile plaques enriched in Aβ peptides (Jack et al., 2024; Cheng et al., 2024). Collectively, these features underscore the urgent need for therapeutic strategies capable of addressing not only symptomatic deficits but also the underlying molecular pathology of the disease.

This framework led to the formulation of the cholinergic hypothesis, proposing that reduced cortical acetylcholine (ACh) levels contribute to cognitive impairment (Bartus et al., 1982). Acetylcholinesterase (AChE) inhibition, therefore, has been a clinically validated approach for enhancing cholinergic neurotransmission and improving cognition (Giacobini, 2004; Tabet, 2006; Grabowska et al., 2025). However, isolated cholinergic modulation addresses primarily symptomatic deficits and does not significantly alter the course of disease progression (Bartus et al., 1982; Lu et al., 2022; Wang et al., 2021). In this context, multifactorial strategies have emerged that aim to target additional pathological drivers of AD, including oxidative stress, neuroinflammation, and protein aggregation. Among these, oxidative stress plays a central role, accelerating Aβ aggregation, promoting lipid peroxidation, and increasing neuronal vulnerability to excitotoxic injury (Bai et al., 2022). Thus, the combination of cholinergic enhancement with antioxidant activity represents a rational strategy to address the multifactorial nature of AD (Weinstock, 2025). Representative AChE inhibitors include the reversible inhibitor donepezil (Li et al., 2018) and the earlier drug tacrine (Eagger et al., 1991). Notably, tacrine was discontinued clinically due to hepatotoxicity and limited tolerability, underscoring the need for safer, multifunctional cholinesterase-targeting scaffolds. Such historical perspective highlights the necessity for design strategies that extend beyond purely cholinergic modulation and integrate complementary neuroprotective mechanisms.

**SCHEME 1:**
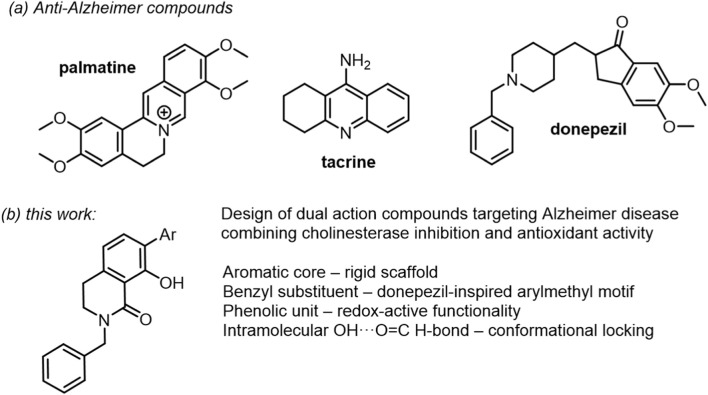
**(a)** Representative acetylcholinesterase inhibitors **(b)** General structure of the 7-aryl tetrahydroisoquinoline derivatives investigated in this study, highlighting key design elements.

Structurally, effective AChE inhibitors generally feature rigid, aromatic-rich frameworks capable of engaging the narrow, aromatic active-site gorge. As illustrated in Scheme 1a, classical inhibitors such as donepezil and tacrine, as well as natural isoquinoline alkaloids like palmatine (Long et al., 2019), employ extended π-systems and cationic or heteroatom-rich motifs that facilitate π–π stacking, cation–π interactions, and polar contacts within the enzyme cavity (Cheng et al., 2017; Reynoso-García et al., 2025). Conformational rigidity and proper surface presentation of aromatic moieties remain critical for optimal engagement of conserved aromatic residues within the AChE gorge.

From a drug-discovery standpoint, the design and synthesis of conformationally defined, polyfunctional heterocycles remain a significant challenge, as molecular rigidity and three-dimensional architecture exert a profound influence on target engagement, selectivity, and overall pharmacological performance. In this context, rigid nitrogen-containing scaffolds inspired by alkaloid architectures have proven particularly valuable (Wetzel et al., 2011). In previous work, our group has demonstrated that such frameworks can be efficiently assembled and strategically functionalized to present well-defined three-dimensional arrangements, (Parra et al., 2017; Parra et al., 2016; Bradshaw et al., 2013a; Bradshaw et al., 2013b), thereby enhancing ligand–target complementarity and binding selectivity in complex biological systems (Lawson et al., 2018).

Building upon this structural foundation, we designed a library of 7-aryl tetrahydroisoquinolines inspired by hybridization of arylmethyl-substituted amine fragments characteristic of donepezil with the isoquinoline core present in palmatine. The scaffold was conformationally constrained through an intramolecular OH···O=C hydrogen bond, which restricts flexibility, promotes effective aromatic surface presentation, and modulates electronic distribution across aryl substituents (Jezierska et al., 2019; Kuhn et al., 2010). These features are expected to (i) stabilize π–π and cation–π interactions within the AChE catalytic gorge, and (ii) tune the redox properties of phenolic functionalities, contributing to antioxidant activity.

Advances in molecular modeling, including docking, binding-energy calculations, and interaction fingerprinting, were employed to assess ligand complementarity with the AChE active site and identify key interactions governing affinity and selectivity (Paggi et al., 2024). Accordingly, the objective of this study was to synthesize a focused set of conformationally locked 7-aryl tetrahydroisoquinolines and evaluate their anti-Alzheimer potential through integrated AChE inhibition, antioxidant assays, and computational modeling, with particular attention to the roles of intramolecular hydrogen bonding and aryl electronic effects.

## 2 Materials and methods

### 2.1 Synthesis of tetrahydroisoquinolines

#### 2.1.1 General procedure A: synthesis of β-keto esters

A solution of DCC (1.1 equiv) in dry CH_2_Cl_2_ (1 mL mmol^-1^) was added dropwise to a stirred solution of Meldrum’s acid (1.0 equiv), the corresponding carboxylic acid (1.0 equiv), and DMAP (1.1 equiv) in dry CH_2_Cl_2_ (5 mL mmol^-1^) at 0 °C under nitrogen. The mixture was stirred for 16 h at 0 °C. The precipitated dicyclohexylurea was removed by filtration and washed with CH_2_Cl_2_.The combined filtrates were washed with 1 M NaHSO_4_, brine, dried (Na_2_SO_4_), and concentrated *in vacuo*. The residue was dissolved in *tert*-butanol (4 mL mmol^-1^) and refluxed under argon for 5 h to induce decarboxylation. After solvent removal, purification by silica gel column chromatography afforded the β-keto esters **1a–f**. Spectroscopic data were consistent with literature (Martínez-Cifuentes et al., 2023).

#### 2.1.2 General procedure B: synthesis of β-keto amide

A mixture of β-keto ester (2.0 equiv) and the appropriate benzylamine derivative (1.0 equiv) in dry toluene (2.5 mL mmol^-1^) was treated with 4-DMAP (0.3 equiv) and heated at 80 °C for 8 h under nitrogen. Reaction progress was monitored by TLC. After cooling, solvent was removed *in vacuo* and the crude product purified by flash chromatography (EtOAc/hexane) to afford β-ketoamides **3a–f**.

#### 2.1.3 General procedure C: synthesis of 7-aryl tetrahydroisoquinolines

β-Ketoamides **3a–f** (1.0 equiv) were dissolved in dry CH_2_Cl_2_ (10 mL mmol^-1^) and treated with Hoveyda–Grubbs 2nd generation catalyst (5 mol%), followed by crotonaldehyde (3.0 equiv). The reaction was heated at reflux for 24 h under nitrogen. After solvent removal, the crude enals were directly dissolved in *i*-PrOH (4 mL mmol^-1^) and treated with Amberlyst-26 (OH^−^ form, 1.0 equiv). The suspension was stirred at room temperature for 48 h. The resin was filtered off, the filtrate washed with saturated NH_4_Cl, extracted with CH_2_Cl_2,_ dried (MgSO_4_), and concentrated. Flash chromatography afforded tetrahydroisoquinolines **5a–f**.

### 2.2 Acetylcholinesterase inhibition assay

AChE inhibition was measured using the Ellman colorimetric method (Cortés et al., 2022). Electric eel acetylcholinesterase (Type VI-S, Sigma) was used at 0.2 U mL^-1^ in Tris–HCl buffer (50 mM, pH 8.0). DTNB was prepared at 0.3 mM and acetylthiocholine iodide at 0.5 mM. Test compounds were dissolved in DMSO and diluted in buffer (final DMSO <1%). The reaction was initiated by addition of substrate and monitored at 405 nm at 37 °C for 30 min using a microplate reader. Galantamine was used as positive control. IC_50_ values (µM) were obtained by nonlinear regression from dose–response curves (GraphPad Prism), expressed as mean ± SD of three independent experiments.

### 2.3 Molecular docking

Docking simulations were performed for compounds **5a–f**, as well as for galantamine, tacrine and donepezil, using the Schrödinger Maestro suite (v11.8) (Schrödinger, 2018). Ligands were prepared using LigPrep, and energy minimization was conducted with the OPLS3e force field. The crystallographic structure of *Torpedo californica* acetylcholinesterase (TcAChE, PDB ID: 1DX6 (Greenblatt et al., 1999) was obtained from the Protein Data Bank (Westbrook, 2017). For tacrine and donepezil, the co-crystallized structures of *Torpedo californica* acetylcholinesterase (PDB IDs: 1ACJ and 1EVE, respectively) (Harel et al., 1993; Kryger et al., 1999) were employed. Protein preparation was carried out using the Protein Preparation Wizard. Water molecules within the active site were removed, and protonation states were assigned at physiological pH (7.4). The simulation box was defined as a 26 Å cubic enclosure. The grid centroid was assigned to the putative catalytic site of the enzyme, based on the positions of the catalytic residues and the bound of galantamine, tacrine or donepezil in the corresponding crystal enzyme structures (Greenblatt et al., 1999; Harel et al., 1993; Kryger et al., 1999). Docking was performed using the Induced Fit Docking protocol followed by Glide XP rescoring (Sherman et al., 2006; Friesner et al., 2006). Binding poses with RMSD <1.0 Å were retained. Galantamine docking was validated against its crystallographic pose. Complexes were visualized using PyMOL and VMD (DeLano, 2002).

### 2.4 Antioxidant assay

#### 2.4.1 Oxygen radical absorbance capacity

The ORAC assay was performed as previously described (Soto et al., 2019). Fluorescein (8 nM), AAPH (75 mM), and samples were prepared in phosphate buffer (75 mM, pH 7.0). Samples (25 μL) were mixed with fluorescein (100 μL) and incubated at 37 °C for 30 min. AAPH (75 μL) was then added, and fluorescence was recorded every minute for 120 min (λ_ex 485 nm, λ_em 520 nm). Results were expressed as µmol Trolox equivalents per µmol compound (µmol TE/µmol).

### 2.5 Computational calculations

Calculations were achieved using the Gaussian 09 software (Frisch et al., 2009), at density functional theory (DFT) M06–2X/6–311+G(d,p) level. No imaginary vibrational frequencies were found at the optimized geometries, indicating that they are the true minimal of the potential energy surface. The calculated thermodynamic parameters were obtained following previous methodology (Martínez-Cifuentes et al., 2023; Monroy-Cárdenas et al., 2024):

$$\text{Bond dissociation enthalpy }\left(\text{BDE}\right):H\left({\text{RO}}^{•}\right)+H\left(H^{•}\right)‐H\left(\text{ROH}\right)$$

$$\text{Ionization pontential }\left(\text{IP}\right):H\left({\text{ROH}}^{•+}\right)+H\left(e^{‐}\right)‐H\left(\text{ROH}\right)$$

where H(RO^•^), H(ROH), and H(ROH^•+^) correspond to the enthalpies of the neutral radical and the neutral and radical cation H(RO^−^) of the compounds, while H(e^−^) and H(H^•^) correspond to enthalpies of the electron (Zheng et al., 2021) and radical hydrogen.

### 2.6 Statistical analysis

Data are presented as mean ± standard error of the mean (SEM). Statistical analyses were performed using one-way ANOVA followed by Tukey’s post hoc test, with p < 0.05 considered statistically significant. Curve fitting and potency calculations (IC_50_ or pIC_50_) were conducted using GraphPad Prism 8 (GraphPad Software Inc., United States) and SigmaPlot 12.0 (Systat Software Inc., United States).

## 3 Results and discussion

### 3.1 Chemistry

The synthetic approach developed in this study was conceived not only to access 7-aryl tetrahydroisoquinolines (7-Ar-THIQs), but also as a structure-guided strategy to generate conformationally restricted scaffolds with tunable electronic properties and potential dual neuroprotective activity. A defining feature of the target molecules is their propensity to form a persistent intramolecular O–H···O=C hydrogen bond between the phenolic moiety and the amide carbonyl. This interaction stabilizes a quasi-planar conformation that is expected to reduce conformational entropy, enhance exposure of the aromatic surface, and favor dispersion-driven π–π interactions, properties that are mechanistically relevant for binding within the aromatic-rich gorge of acetylcholinesterase (AChE) (Lawson et al., 2018; Jezierska et al., 2019; Kuhn et al., 2010). The exclusive exploration of the ortho substitution pattern was structurally motivated by the need to enable intramolecular O–H···O=C hydrogen bond formation. Only the ortho arrangement provides the spatial proximity required between the phenolic hydroxyl group and the C1 amide carbonyl of the tetrahydroisoquinoline core, thereby enforcing conformational locking and controlled presentation of the aromatic surface. In contrast, meta or para substitution would not be expected to support this intramolecular interaction and were therefore not pursued in the present design strategy. In parallel, systematic electronic modulation of the 7-aryl substituent (electron-donating, neutral, or electron-withdrawing) provides a means to tune aromatic polarizability, π-electron density, and redox behavior, parameters that are directly relevant to both AChE inhibition and antioxidant activity (Jezierska et al., 2019; Kuhn et al., 2010). β-Ketoesters 1a–f are known compounds prepared according to literature procedures [30], whereas β-ketoamides **3a**–**f** and the final tetrahydroisoquinoline derivatives **5a**–**f** are novel compounds reported here for the first time. The intermediate enals **4a**–**f** were not isolated due to their instability under chromatographic conditions.

**SCHEME 2:**
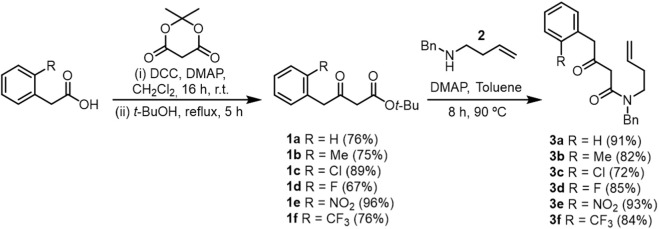
Synthesis of β-ketoamides.

Guided by this structural rationale, the synthetic sequence began with the preparation of key β-ketoamide intermediates **3a–f** by aminolysis of β-ketoesters **1a–f** (prepared from Meldrum’s acid) with benzylamine derivatives **2** under conventional β-ketoester–amine coupling conditions (Scheme 2). This step enabled early introduction of the aryl substituent, allowing systematic variation across the library. The β-ketoamides were isolated in good purity and served as precursors for the tetrahydroisoquinoline core.

**SCHEME 3:**
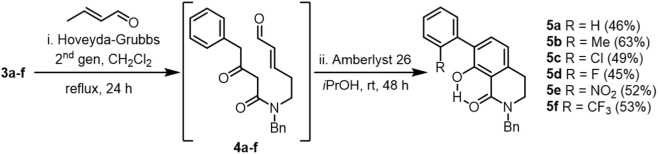
Synthesis of THIQ via Cross Metathesis/Tandem Cyclization Reaction.

The essential enal moieties (**4a–f**) required for intramolecular Michael addition were installed via cross-metathesis between β-ketoamides **3a–f** and crotonaldehyde using the second-generation Grubbs–Hoveyda catalyst. Although minor Michael-type byproducts were observed, the crude enals were directly used in the subsequent cyclization step due to their instability under chromatographic conditions, in line with previous experience (Parra et al., 2017). Cyclization under basic conditions promoted a tandem intramolecular Michael addition followed by Robinson-type annulation, tautomerization, and aerobic oxidation (Keerthana et al., 2024), furnishing tetrahydroisoquinolines **5a–f** as the major products (Scheme 3).

The methodology proved robust across both electron-rich and electron-deficient aryl systems, with yield variations reflecting substrate-dependent intermediate stability and the higher susceptibility of electron-poor derivatives to undergo competing pathways, as commonly observed in Michael/annulation cascade reactions (Spitzbarth and Eelkema, 2023; Tietze, 1996). Importantly, all THIQ products exhibited a characteristic downfield phenolic singlet in the ^1^H NMR spectrum (≈13 ppm), diagnostic of a strong intramolecular O–H···O=C hydrogen bond. This spectroscopic signature confirms the intended conformational preorganization of the scaffold and provides a structural basis for correlating aryl electronics and conformational locking with AChE inhibition, antioxidant capacity (ORAC), and *in silico* binding behavior.

### 3.2 AChE inhibition assays

The acetylcholinesterase inhibitory profile of the 7-substituted tetrahydroisoquinoline series reveals a coherent structure–activity relationship governed primarily by the electronic nature of the aryl substituent and its consequences for conformational locking through intramolecular hydrogen bonding. Across the series (Table 1), IC_50_ values range from 33.09 to 54.27 µM, weaker than the reference inhibitor galantamine (IC_50_ = 4.67 µM), yet highly systematic in their dependence on substituent electronics. Compounds bearing electron-donating or weakly electron-withdrawing groups (H, Me, Cl) exhibit the highest activities (IC_50_ = 33–48 μM; pIC_50_ = 4.48–4.32), whereas strongly electron-withdrawing substituents (F, NO_2_, CF_3_) consistently produce the lowest potencies (IC_50_ ≈ 53–54 μM; pIC_50_ ≈ 4.27–4.26). This pattern is structurally meaningful: electron-withdrawing groups decrease the electron density and polarizability of the 7-aryl ring, thereby attenuating the dispersion-dominated π–π stacking interactions that dominate ligand recognition in aromatic-rich protein cavities, in agreement with recent large-scale statistical analyses of aromatic contacts in ligand–protein complexes (Brylinski, 2018). Conversely, electron-rich systems enhance π-electron density and polarizability, improving their capacity to engage in stabilizing π–π and CH–π contacts at protein–ligand interfaces, as shown by quantum-mechanical and statistical analyses of substituent-dependent effects on aromatic interactions (Wheeler and Houk, 2009; Parrish and Sherrill, 2014; Jiménez-Moreno et al., 2015).

**TABLE 1:** Chemical shifts of the OH proton as a function of the electronic properties of the substituents.

Compound	Substituent	AChE inhibition IC_50_ (µM)	pIC_50_	δ(OH) (^1^H NMR, ppm)
5a	H	40.91 ± 1.57 ^*a*^	4.388	13.18
5b	Me	33.09 ± 0.69 ^*b*^	4.480	12.90
5c	Cl	48.06 ± 1.20 ^*c*^	4.318	12.99
5d	F	53.45 ± 0.81 ^*c*^	4.272	13.09
5e	NO_2_	52.82 ± 0.29 ^*c*^	4.277	13.05
5f	CF_3_	54.27 ± 0.96 ^*c*^	4.265	12.87
Galantamine*	—	4.67 ± 0.14 ^*d*^	5.331	—

* Used as positive control; Values having different superscripts differ significantly (p < 0.05).

Notably, no direct correlation was observed between the phenolic OH chemical shift and AChE inhibitory potency across the series (*R* ^2^ ≈ 0), indicating that variations in intramolecular hydrogen-bond strength do not govern binding affinity. Thus, δ(OH) reports primarily on scaffold preorganization rather than on the availability of the phenolic proton for direct enzyme binding. Instead, the substituent-dependent modulation of the aromatic π-surface appears to be the dominant determinant of activity. A second structural element that strongly influences activity is the formation of a favored intramolecular OH···O=C hydrogen bond, experimentally supported by the deshielded phenolic singlets observed in the ^1^H NMR spectra (δ = 12.87–13.18 ppm).

As a result, the scaffold is biased toward a preorganized geometry in which the 7-aryl π-surface is optimally presented for π-driven recognition. Subtle substituent-dependent variations in OH chemical shift suggest minor differences in intramolecular H-bond strength, yet these do not correlate directly with potency, reinforcing that π-surface modulation, rather than H-bond rearrangement, governs affinity in this scaffold. The intermediate activity of the unsubstituted phenyl analogue (IC_50_ = 40.91 µM, pIC_50_ = 4.39) is fully consistent with this model: it offers a neutral, planar, and electronically balanced aromatic surface that avoids the destabilizing torsional or polar distortions observed in strongly electron-withdrawing derivatives. The most active compound, the 7-methyl analogue (IC_50_ = 33.09 µM, pIC_50_ = 4.48), exemplifies how modest electron donation enhances aromatic polarizability without disrupting conformational locking, thereby improving π-driven recognition within the gorge.

Taken together, the SAR analysis suggests that (i) the π-electron density and polarizability of the 7-aryl ring are the primary contributors to AChE affinity, (ii) all compounds rely on a conformationally locked intramolecular H-bond that enforces scaffold preorganization and modulates desolvation, thereby biasing recognition toward π-driven interactions, and (iii) electron-withdrawing substituents reduce binding mainly by diminishing the effectiveness of dispersion-based aromatic contacts. Although these SAR observations provide preliminary insights into the influence of aryl substitution, the modest activity differences (≤2-fold) indicate that the findings should be considered exploratory rather than definitive. It is important to note, however, that classical models of aromatic interactions, including the Hunter–Sanders framework (Hunter and Sanders, 1990; Wheeler, 2025), indicate that electron-withdrawing substituents may reduce quadrupole-driven electronic repulsion and, in certain geometrical arrangements (e.g., edge-to-face stacking), can favor specific aromatic interaction modes. Therefore, substituent effects in π–π interactions are not governed exclusively by π-electron density but also by electrostatic components and relative ring orientation. In the confined environment of the AChE gorge, where stacking geometry and steric complementarity are strongly constrained, the net effect observed in this series likely reflects the combined influence of dispersion forces, electrostatics, and packing efficiency.

Although the catalytic gorge of AChE is predominantly aromatic and hydrophobic, it also contains strategically positioned polar residues and backbone-exposed heteroatoms capable of contributing to local electrostatic microenvironments. In this context, strongly electron-withdrawing substituents may increase the local electrostatic character of the aryl surface and alter desolvation energetics upon binding. Such effects could introduce unfavorable electrostatic or entropic contributions to the overall binding free energy, particularly when optimal π–π complementarity is not achieved. Within the limited substituent set evaluated, electron-donating or weakly withdrawing groups appear to favor improved affinity; however, given that only one electron-donating substituent (Me) was examined, this trend should be interpreted cautiously and considered preliminary pending evaluation of a broader electronic range.

Beyond their effects on AChE, the structural features governing aryl electronics, aromatic polarizability, and intramolecular hydrogen bonding also appear to play key roles in antioxidant behavior. Substituent-dependent modulation of aryl electronics and phenoxyl radical stabilization is expected to influence radical-quenching capacity, while the conformationally locked phenolic OH remains capable of participating in hydrogen-atom transfer pathways under oxidative conditions. These parallel structure–function relationships suggest that modifications that optimize π-electron density for AChE binding may simultaneously influence ORAC performance, providing an integrated framework to evaluate the dual neuroprotective potential of this scaffold.

### 3.3 Molecular docking analysis

**FIGURE 1:**
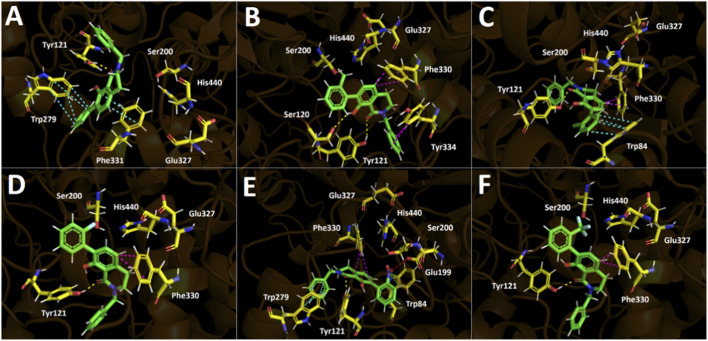
Predicted binding modes and intermolecular interactions of compounds 5a–f within the acetylcholinesterase catalytic gorge. Hydrogen bonds are shown in yellow, π–π interactions in cyan, T-shaped interactions in magenta, π–cation interactions in green, and salt bridges in red. Panels **(A–F)** correspond to compounds 5a–f, respectively.

Molecular docking was employed to examine how the conformationally locked 7-aryl tetrahydroisoquinoline scaffold is accommodated within the aromatic gorge of acetylcholinesterase and to rationalize the experimentally observed structure–activity relationships. The predicted binding poses for compounds **5a–f** reveal a highly conserved binding mode, in which all ligands occupy the same region of the catalytic gorge and interact with a common set of aromatic residues that define the π-rich lining of the active site (Figure 1). The corresponding docking scores, obtained using Glide, are summarized in Table 2.

**TABLE 2:** Docking scores (Glide) of compounds **5a–f** and galantamine at the acetylcholinesterase active site.

Compound	Binding energy (kcal/mol)
5a	−10.810
5b	−10.924
5c	−10.249
5d	−10.462
5e	−10.133
5f	−11.498
Galantamine	−11.749

As shown in Table 2, all derivatives display very similar docking scores (−10.1 to −11.5 kcal/mol), consistent with their experimentally observed micromolar AChE inhibition. The relatively narrow energetic window mirrors the subtle potency differences across the series and is consistent with a conserved binding mode, where small changes in aromatic packing and hydrophobic complementarity, not major pose rearrangements, are expected to drive modest activity shifts within the uncertainty limits of docking/scoring functions (Paggi et al., 2024; Warren et al., 2006). Across the entire series, the lactam carbonyl establishes a conserved hydrogen bond with Tyr121, anchoring the tetrahydroisoquinoline core in a fixed orientation within the gorge. However, this interaction does not differentiate potency between derivatives. In contrast, recognition is dominated by aromatic and hydrophobic contacts with Trp84, Trp279, and Phe330/Phe331, which form the π-rich walls of the AChE catalytic gorge. These interactions are fully consistent with the experimentally derived SAR showing that aryl electronic properties and π-surface complementarity govern AChE inhibition in this scaffold. Unlike classical acetylcholinesterase inhibitors such as donepezil, the present scaffold lacks a permanently protonated tertiary amine capable of establishing strong cation–π interactions within the catalytic anionic site. This structural difference may partly explain the moderate micromolar potency observed, as binding in this series appears to rely predominantly on π–π stacking and hydrophobic complementarity rather than electrostatic anchoring.

Compound 5a, which lacks substitution on the aryl ring, adopts an inverted orientation relative to derivatives 5b–f (Figure 1A). In contrast, compounds 5b–f bear substituents at the ortho position of the aryl ring. The absence of steric and stereoelectronic bias in 5a likely permits increased torsional freedom around the aryl–isoquinoline bond, enabling alternative docking solutions of comparable energy. Conversely, ortho substituents in 5b–f introduce localized steric demand and stereoelectronic constraints proximal to the biaryl junction, restricting conformational space and favoring a conserved orientation of the aryl vector within the catalytic gorge. Thus, the inverted conformation observed for 5a is consistent with reduced ortho steric control rather than with electronic modulation alone. (Figures 1B–F). The 7-methyl derivative **5b**, the most potent compound in the series, shows optimal alignment of its aryl ring against Phe330 and Tyr334, maximizing aromatic surface complementarity. The chloro derivative **5c** retains favorable π-stacking with Trp84, whereas the fluoro, nitro, and trifluoromethyl derivatives (**5d–f**) display progressively less optimal packing due to reduced polarizability or steric and electronic perturbation of the aryl ring. Although compound **5e** (NO_2_) engages in additional polar interactions in the docking model, these do not translate into improved inhibitory potency, indicating that binding in this series is not driven by the number of directional hydrogen bonds or electrostatic contacts but rather by the quality of π-driven and hydrophobic complementarity within the aromatic gorge. This trend is consistent with large structural analyses showing that hydrophobic interactions often dominate high-efficiency ligand binding and that aromatic π-stacking contacts contribute significantly to protein–ligand recognition, even in the presence of hydrogen bonds (de Freitas and Schapira, 2017). Although hydrophobic and π interactions dominate binding in this series, conserved polar residues in the AChE gorge indicate that electrostatic complementarity also modulates affinity. Strongly electron-withdrawing substituents may reduce dispersion interactions and introduce unfavorable electrostatic or desolvation effects. Therefore, binding arises from a balance between π-driven dispersion forces and electrostatic contributions to the overall free energy.

**FIGURE 2:**
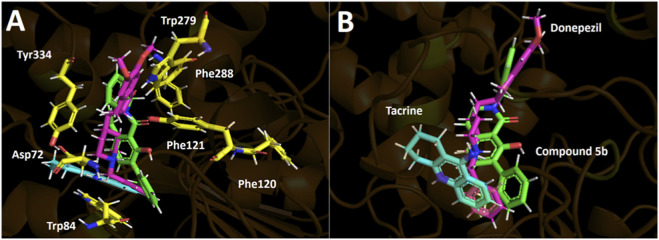
Structural superimposition of the most active compound 5b (green) and the established inhibitors tacrine (cyan) and donepezil (magenta) within the catalytic site of acetylcholinesterase (TcAChE). **(A)** Compound 5b, tacrine, and donepezil depicted in the presence of the amino acid residues mediating their intermolecular interactions. **(B)** Overlay of compound 5b, tacrine, and donepezil in the absence of the interacting amino acid residues.

Figure 2 shows the structural superposition of the most active compound of series (5b) with tacrine and donepezil. The binding poses of tacrine and donepezil were obtained from the crystal structures 1ACJ (Harel et al., 1993) and 1EVE (Kryger et al., 1999), respectively, deposited in the RCSB Protein Data Bank.

Tacrine forms a salt bridge with Asp72, π–π interactions with Trp84 and Phe330, and a hydrogen bond with His440. Donepezil exhibits π–π interactions with Trp84 and Trp279, a salt bridge with Asp72, and π–cation interactions with Phe330 and Tyr334. Compound 5b shows a binding mode more similar to donepezil than tacrine (Figure 2B). Although Phe330 and Tyr334 are involved in both cases, donepezil forms salt bridges, whereas 5b establishes T-shaped interactions. Despite the weaker nature of these interactions, 5b may compensate through hydrogen bonds with Ser120 and Tyr121 (Figure 1).

Although the present study does not include meta- or para-substituted derivatives, positional effects can be tentatively considered. Para substitution would be expected to modulate electronic properties of the aromatic ring without significantly altering torsional bias at the biaryl junction and therefore may not substantially perturb the conformational behavior observed for the unsubstituted analogue. In contrast, meta substitution could introduce intermediate steric and electronic effects whose conformational consequences cannot be predicted with certainty and would require dedicated evaluation. Future exploration of these positional variants will be necessary to establish whether their preferred orientation more closely resembles the ortho-substituted derivatives or the unsubstituted scaffold.

The docking results reinforce the NMR-based mechanistic interpretation, indicating that the intramolecular hydrogen bond preorganizes the scaffold and orients the 7-aryl ring in alignment with the π-rich topology of the AChE gorge, thereby facilitating aromatic contacts. Conformational restriction is a recognized strategy in medicinal chemistry to promote bioactive conformations and reduce entropic penalties upon binding (Fang et al., 2014), and reduced ligand flexibility has been correlated with improved binding energetics and interaction complementarity (DeLorbe et al., 2009). In this context, aromatic interactions, particularly π–π stacking, are key determinants of molecular recognition in aromatic-rich binding pockets (Zhu et al., 2021).

Accordingly, all ligands adopt nearly identical docking orientations, with the 7-aryl ring positioned against Trp84 and Phe330 and the lactam core stabilized by a conserved hydrogen bond with Tyr121. The absence of correlation between δOH and AChE potency further indicates that the intramolecular hydrogen bond functions primarily as a structural preorganization element rather than as a direct pharmacophoric interaction with the enzyme. From a medicinal chemistry perspective, the 7-aryl vector represents a strategic site for further optimization. Binding in this series appears to be dominated by π–π stacking and hydrophobic complementarity within the aromatic-rich catalytic gorge. (Wheeler, 2013). Therefore, introduction of highly polar or excessively labile substituents along this vector could disrupt favorable desolvation energetics and attenuate hydrophobic interactions with residues such as Trp84 and Phe330. Conversely, carefully designed substituents capable of forming additional directional interactions (e.g., hydrogen bonds with peripheral or catalytic residues) could enhance binding enthalpy and potentially improve pharmacodynamic efficiency. It should also be considered that ortho substitution contributes to conformational bias in this scaffold; therefore, excessive flexibility or steric perturbation may compromise the preorganized geometry enforced by the intramolecular OH···O=C hydrogen bond. Accordingly, future analogue design should balance conformational control, hydrophobic complementarity, and physicochemical modulation to optimize both binding efficiency and drug-like properties.

Overall, the docking analysis supports a conserved, π-dominated binding mode in which electronic and steric modulation of the 7-aryl substituent fine-tunes how effectively the aromatic surface engages the AChE gorge, in excellent agreement with the experimentally observed structure–activity relationships.

### 3.4 Antioxidant evaluation and computational analysis

The antioxidant capacity of compounds **5a**–**5f** was evaluated using the ORAC assay, with results expressed as µmol Trolox equivalents per µmol of compound (Table 3).

**TABLE 3:** ORAC values of 7-aryl tetrahydroisoquinolines expressed as µmol Trolox equivalents per µmol compound.

Compound	Substituent	ORAC (µmol Trolox/µmol compound)
5a	H	0.036 ± 0.003 ^*a*^
5b	Me	0.038 ± 0.001 ^*a*^
5c	Cl	0.049 ± 0.001 ^*b*^
5d	F	0.047 ± 0.001 ^*b*^
5e	NO_2_	0.033 ± 0.001 ^*c*^
5f	CF_3_	0.032 ± 0.003 ^*c*^
Ascorbic acid*	—	0.480 ± 0.040 ^*d*^

* Used as positive control; Values having different superscripts differ significantly (p < 0.05).

Compounds **5a**–**5f** exhibit significantly lower antioxidant capacities compared to the reference compound, ascorbic acid, whose activity was approximately one order of magnitude higher than that of the tested derivatives. This difference suggests that, although the compounds display some antioxidant activity, their effectiveness is considerably limited.

When comparing compounds **5a**–**5f** among themselves, a modest influence of the substituent type on antioxidant capacity is observed: compounds **5c** and **5d** showed the highest values in series (0.049 and 0.047 µmol Trolox/µmol, respectively), which may be attributed to inductive effects and radical-stabilizing interactions within the molecular framework. In contrast, derivatives bearing strongly electron-withdrawing groups such as **5e** and **5f** exhibited the lowest activities (0.033 and 0.032 µmol Trolox/µmol, respectively), possibly due to a reduction in electron density, leading to decreased radical stabilization ability.

The relatively low ORAC values, compared to both the reference compound and typical phenolic antioxidants, suggest that the antioxidant activity of this series may not be centered on the O–H bond. A structural factor that may explain the variation in antioxidant capacity is the presence of a favorable intramolecular OH···O=C hydrogen bond. It is known that intramolecular hydrogen bonds can modulate the O–H bond dissociation enthalpy by stabilizing specific conformations and altering the electronic environment of the phenolic group (Rohman et al., 2023). Recent theoretical studies on polyphenolic antioxidants have shown that these conformational and electronic effects mediated by hydrogen bonding directly influence phenoxyl radical stability and hydrogen atom transfer (HAT) efficiency (Vlocskó et al., 2025).

The antioxidant activity of small molecules is primarily governed by their ability to neutralize reactive radicals through HAT and, to a lesser extent, single-electron transfer (SET) mechanisms. Thermochemical descriptors such as bond dissociation enthalpies (BDEs) and ionization potentials (IPs) are therefore widely used to evaluate the feasibility of these pathways at the molecular level. Lower BDE values favor HAT processes, whereas lower IP values indicate a greater propensity for SET-based radical scavenging. Density functional theory calculations at the M06–2X/6–311+G(d,p) level were performed to evaluate (IP), the bond dissociation enthalpy of the phenolic O–H bond (BDE1), and the bond dissociation enthalpy of the benzylic C–H bond of the benzyl substituent (BDE2). The computed values are summarized in Table 4.

**TABLE 4:** Ionization potentials (IP) and bond dissociation enthalpies (BDE, kcal/mol) for the O–H (BDE1) and benzylic C–H (BDE2) bonds.

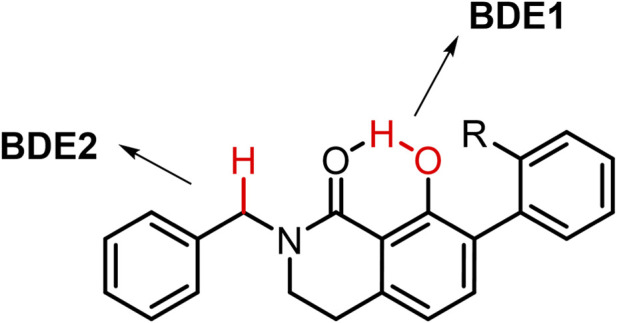				
Compound	Substituent	IP (kcal/mol)	BDE1 (kcal/mol)	BDE2 (kcal/mol)
5a	H	172.5	92.1	80.8
5b	Me	172.9	93.2	78.4
5c	Cl	173.6	91.8	80.8
5d	F	174.9	89.9	79.6
5e	NO_2_	172.5	91.3	78.3
5f	CF_3_	168.6	90.8	76.9

The relatively high IP values observed across series (167.8–174.1 kcal/mol), when compared with the corresponding BDE values, indicate that SET-type mechanisms are thermodynamically disfavored, supporting a predominantly HAT-driven antioxidant behavior. Notably, the calculated BDE2 values are consistently 10–15 kcal/mol lower than the corresponding BDE1 values, indicating that benzylic hydrogen abstraction is thermodynamically more favorable than homolytic cleavage of the phenolic O–H bond.

This energetic preference can be rationalized by the presence of a favored intramolecular OH···O=C hydrogen bond, experimentally evidenced by strongly deshielded phenolic ^1^H NMR signals. Intramolecular hydrogen bonding stabilizes the phenolic O–H bond, effectively increasing its BDE and biasing the system against direct hydrogen atom transfer from the O–H group. In contrast, the benzylic CH_2_ position remains electronically activated through conjugation with the adjacent aromatic ring, providing a thermodynamically accessible hydrogen-donor site whose resulting radical is efficiently stabilized.

Importantly, this energetic bias does not imply that phenolic moiety is irrelevant to antioxidant activity. Rather, the calculations suggest a dual-site HAT framework in which the benzylic CH_2_ group acts as the preferred hydrogen donor, while the phenolic unit functions as an electronically tunable modulator of radical stabilization. Similar behavior has been reported for xanthone- and flavonoid-type antioxidants, where benzylic or allylic C–H bonds can compete with, or even dominate over, phenolic O–H groups in HAT processes despite the presence of classical phenolic functionalities (Zheng et al., 2021; Vo et al., 2019).

Within this framework, the experimentally observed ORAC trends can be rationalized by substituent-dependent modulation of aryl electronic properties. Variations in aromatic substitution influence the delocalization and stability of both benzylic and phenoxyl radicals, thereby affecting overall radical-quenching efficiency even when the primary hydrogen abstraction event occurs at the C–H site.

Thus, intramolecular hydrogen bonding suppresses direct O–H hydrogen donation while preserving the phenolic group as an electronic regulator of antioxidant performance. Overall, computational analysis supports a predominantly HAT-based antioxidant mechanism characterized by benzylic hydrogen abstraction within a conformationally preorganized scaffold. The combined effects of intramolecular hydrogen bonding and aryl electronic modulation provide a coherent thermochemical explanation for the ORAC behavior of this series and reinforce the integrated structure–function model underlying their dual neuroprotective activity.

## 4 Conclusion

This study introduces a new family of conformationally locked 7-aryl tetrahydroisoquinoline derivatives, rationally designed as multitarget agents aimed at key pathological processes in Alzheimer’s disease. The main innovation of this series lies in its molecular architecture, which features a stable intramolecular O–H···O=C hydrogen bond that restricts conformational flexibility and promotes a quasi-planar spatial arrangement of the aromatic system. This structural preorganization not only enhances π–π interactions with conserved aromatic residues within the acetylcholinesterase catalytic gorge but also modulates the electronic properties of the aryl ring, influencing both enzyme affinity and antioxidant capacity.

The compounds display moderate inhibitory activity against AChE in the micromolar range, with a well-defined structure–activity relationship (SAR) governed by the electronic nature of the aryl substituents. In parallel, their antioxidant capacity, although modest compared to standards such as ascorbic acid, is also structure-dependent, supported by DFT calculations showing a thermodynamic preference for hydrogen atom transfer (HAT) from benzylic C–H bonds rather than from phenolic O–H groups. This dual behavior, as cholinergic inhibitors and free radical scavengers, highlights the potential of these molecules as neuroprotective candidates with complementary mechanisms of action. Although further optimization is needed to improve biological potency, the consistent experimental and computational findings, along with the synthetic versatility of the THIQ core, establish this scaffold as a valuable starting point for the development of compounds with improved potency, selectivity, and drug-like properties. The moderate potency observed relative to classical cholinesterase inhibitors underscores the exploratory nature of this scaffold and highlights opportunities for future structural refinement.

## Data Availability

The data presented in the study are included in the article/Supplementary Material, further inquiries can be directed to the corresponding authors.
