# Supplementary material for: Conformationally locked 7-aryl tetrahydroisoquinolines as dual acetylcholinesterase inhibitors and antioxidants: role of intramolecular hydrogen bonding and aryl electronics
Source: Front Pharmacol. 2026 Apr 8;17:1797738. doi: 10.3389/fphar.2026.1797738 (PMC13099125; doi:10.3389/fphar.2026.1797738)
Supplement: Supplementary file 1 [file Supplementaryfile1.docx]

**Conformationally Locked 7-Aryl Tetrahydroisoquinolines as Dual Acetylcholinesterase Inhibitors and Antioxidants: Role of Intramolecular Hydrogen Bonding and Aryl Electronics**

Valentina Ríos ^1^, Camila Linares-Pipón ^1^, Alejandro Castro-Álvarez ^2^, Ben Bradshaw ^3^, Javier Romero-Parra ^4^, Mauricio A. Cuellar ^5^, Maximiliano Martínez-Cifuentes ^1,^* and Claudio Parra ^1,^*

^1^ Departamento de Química Orgánica, Facultad de Ciencias Químicas, Universidad de Concepción, Edmundo Larenas 129, Concepción 4070371, Chile.

^2^ Departamento de Ciencias Preclínicas, Facultad de Medicina, Universidad de La Frontera, Av. Francisco Salazar 01145, Temuco 4780000, Chile.

^3^ Laboratori de Química Organica, Facultat de Farmacia, IBUB, Universitat de Barcelona, 08028 Barcelona, Spain.

^4^ Departamento de Química Orgánica y Fisicoquímica, Facultad de Ciencias Químicas y Farmacéuticas, Universidad de Chile, Santiago 6640022, Chile.

^5^ Centro de Investigación Farmacopea Chilena, Escuela de Química y Farmacia, Facultad de Farmacia, Universidad de Valparaíso, Av. Gran Bretaña 1093, Valparaíso 2360102, Chile.

**Supporting Information**

**Table of Contents**

Experimental and NMR data of compounds **1**-**8 S1 – S8**

Copies of 1H- and 13C-NMR spectra **S9 – S32**

Copies of MS spectra **S33 – S36**

**Experimental**

**General**. All reactions were carried out under an argon atmosphere with dry, freshly distilled solvents under anhyd conditions. Analytical thin-layer chromatography was performed on SiO_2_ (Merck silica gel 60 F_254_), and the spots were located with 1% aqueous KMnO_4_. Chromatography refers to flash chromatography was carried out on SiO_2_ (SDS silica gel 60 ACC, 35-75 µm, 230-240 mesh ASTM). Drying of organic extracts during workup of reactions was performed over anhyd MgSO_4_ except where stated otherwise. Evaporation of the solvent was accomplished with a rotatory evaporator. NMR spectra were recorded in CDCl_3_ on a Varian VNMRS 400. Chemical shifts of ^1^H and ^13^C NMR spectra are reported in ppm downfield (δ) from Me_4_Si.

**General information**

All reactions were carried out an argon atmosphere with dry, freshly distilled solvents under anhydrous conditions. Analytical thin-layer chromatography was performed on SiO_2_ (Merck silica gel 60 F_254_), and the spots were located with 1 % aqueous KMnO_4_. Chromatography refers to flash chromatography was carried out on SiO_2_ (SDS silica gel 60 ACC, 35-75 µm, 230-240 mesh ASTM). Drying organic extracts during the workup of reactions was performed over anhydrous Na_2_SO_4_. Evaporation of the solvent was accomplished with a rotatory evaporator. NMR spectra were recorded in CDCl_3_ on a Bruker 400. Chemical shifts of ^1^H and ^13^C spectra are reported in ppm downfield (δ) from Me_4_Si.

*N*-Benzylbut-3-en-1-amine (2). To a solution of 4-bromobutene (0.8 mL, 7.4 mmol, 1 equiv) in acetonitrile (15 mL) was added benzylamine (2.4 mL, 22.2 mmol, 3 equiv). The mixture was heated at reflux for 4 h. After cooling to room temperature, a saturated NaHCO_3_ solution (15 mL) was added and the organic layer was extracted with diethyl ether (3 x 15 mL). The combined extracts were dried (Na_2_SO_4_) concentrated *in vacuo* and purified by column chromatography (0→10→25→50% EtOAc/hexane) to give 2 as a yellow oil in 97% yield (1.16 g); ^1^H NMR (400 MHz, COSY) δ 2.28 (qt, J = 8.0, 6.8, 2.4, 1.2 Hz, 2H, H-2), 2.70 (t, J = 6.8 Hz, 2H, H-1), 3.79 (s, 2H, CH_2_Ph), 5.03 (dq, J = 4.0, 2.8, 2.0, 1.2 Hz, 1H, H-4), 5.08 (dq, J = 4.8, 3.2, 1.6 Hz, 1H, H-4) 5.78 (ddt, J= 14.0, 10.4, 6.8 Hz, 1H, H-3) 7.24 (m, 1H, Ph), 7.31 (m, 4H, Ph); ^13^C NMR (100 MHz, HSQC) δ 34.4 (C-2), 48.4 (C-1), 54.0 (CH_2_Ph), 116.4 (C-4), 127.0 (Ph), 128.2 (Ph), 128.5 (Ph), 136.6 (C-3), 140.5 (Ph). HRMS calcd for C_11_H_16_N [M+H]^+^ 162.1277, found 162.1278. Spectral data was identical to that previously reported (10.1021/ol400926p).

***N*-Benzyl-*N*-(But-3-en-1-yl)-3-oxo-4-phenylbutanamide (3a).** To a mixture of *N*-benzylbut-3-en-1-amine (344 mg, 2.13 mmol), *tert*-Butyl 3-oxo-5-phenylpentanoate (1.00 g, 4.26 mmol) in toluene (5.3 mL) was added 4-DMAP (77 mg, 0.636 mmol). The mixture was stirred at 80 ºC under Ar for 8 h. After cooling to room temperature, the solvent was removed *in vacuo*. Purification by column chromatography (0→10→25→50% EtOAc/hexane) gave β-ketoamide **3a** (0.685 g, 91%) as a yellow oil. ***Rotamer A*:** ^1^H NMR (400 MHz, COSY) δ 2.16 (q, *J* = 14.4, 7.2 Hz, 2H, H-2’), 2.16 (q, *J* = 9.2 Hz, 2H, H-1’), 3.50 (s, 2H, H-2), 3.84 (s, 2H, H-4), 4.40 (s, 2H, CH_2_Ph), 5.01 (m, 2H, H-4’), 5.59 (m, 1H, H-3’) 7.09 (m, 1H, Ph), 7.25 (m, 10H, Ph); ^13^C NMR (100 MHz, HSQC) δ 32.6 (C-2’), 46.7 (C-1’), 48.1 (C-2), 50.1 (C-4), 51.9 (CH_2_Ph), 87.9, 117.8 (C-4’), 127,6 (Ar), 133.9 (C-3’), 167.1 (C-3), 202.2 (C-1); ***Rotamer B*:** ^1^H NMR (400 MHz, COSY) δ 2.31 (q, *J*= 7.2 Hz, 2H, H-2’), 2.31 (m, 2H, H-1’), 3.60 (s, 2H, H-2), 3.89 (s, 2H, H-4), 4.54 (s, 2H, CH_2_Ph), 5.01 (m, 2H, H-4’), 5.77 (m, 1H, H-3’), 7.09 (m, 1H, Ph), 7.25 (m, 10H, Ph); ^13^C NMR (100 MHz, HSQC) δ 32.0 (C-2’), 46.0 (C-1’), 47.8 (C-2), 48.2 (CH_2_Ph), 50.0 (C-4), 87.5, 116.9 (C-4’), 127,1 (Ar), 135.1 (C-3’), 166.8 (C-3), 202.1 (C-1); HRMS calcd for C_21_H_24_NO_2_ [M+H]^+^ 322.1802, found 322.1802.

***N*-benzyl-*N*-(but-3-en-1-yl)-3-oxo-4-(*o*-tolyl)butanamide (3b).** To a mixture of *N*-benzylbut-3-en-1-amine (0.098 g, 0,61 mmol), *tert*-Butyl 3-oxo-4-(o-tolyl)butanoate (0,302 g, 1,22 mmol) in toluene (1.5 mL) was added 4-DMAP (23 mg, 0,186 mmol). The mixture was stirred at 80 ºC under Ar for 8 h. After cooling to room temperature, the solvent was removed *in vacuo*. Purification by column chromatography (0→5→10→25% EtOAc/hexane) gave β-ketoamide **3b** (00,167 g, 82%) as a yellow oil. ***Rotamer A*:** ^1^H NMR (400 MHz, COSY) δ 2.16 - 2.38 (m, 2H, H-2’), 2.28 (s, 3H, CH_3_), 3.48 (t, J = 8.0 Hz, 2H, H-1’), 3.61 (s, 2H, H-2), 3.94 (s, 2H, H-4), 4.65 (s, 2H, CH2Ph), 4.83 – 5.13 (m, 2H, H-4’), 5.73 – 5.87 (m, 1H, H-3’) 7.04 – 7.39 (m, 9H, Ph); 13C NMR (100 MHz, CDCl_3_) δ 19.8 (CH3), 32.8 (C-2’), 46.1 (C-1’), 48.4 (C-2), 52.1 (CH_2_Ph), 117.9 (C-4’), 126.4, 127.7, 127.8, 128.1, 128.8, 129.1, 130.1, 135.3, 137.3 (Ph), 134.0 (C-3’), 167.2 (C-3), 202.1 (C-1);; ***Rotamer B*:** ^1^H NMR (400 MHz, COSY) δ 2.16 - 2.38 (m, 2H, H-2’), 2.25 (s, 3H, CH_3_), 3.18 (t, J = 8.0 Hz, 2H, H-1’), 3.52 (s, 2H, H-2), 3.90 (s, 2H, H-4), 4.45 (s, 2H, CH_2_Ph), 5.13 – 4.83 (m, 2H, H-4’), 5.57 – 5.71 (m, 1H, H-3’) 7.04 – 7.39 (m, 9H, Ph); 13C NMR (100 MHz, CDCl3) δ 19.8 (CH3), 32.1 (C-2’), 47.1 (C-1’), 48.5 (C-2), 52.1 (CH2Ph), 117.0 (C-4’), 126.4, 127.7, 127.8, 128.1, 128.8, 129.1, 130.1, 135.3, 137.3 (Ph), 134.0 (C-3’), 167.2 (C-3), 202.1 (C-1). HRMS calcd for C_22_H_25_NO_2_ [M+H]^+^ 335.1919, found 336.1902.

***N*-benzyl-*N*-(but-3-en-1-yl)-4-(2-chlorophenyl)-3-oxobutanamide (3c).** To a mixture of *N*-benzylbut-3-en-1-amine (359 mg, 2.23 mmol), *tert*-Butyl 4-(2-Chlorophenyl)-3-oxobutanoate (570 mg, 4.34 mmol) in toluene (6.0 mL) was added 4-DMAP (82 mg, 0.67 mmol). The mixture was stirred at 80 ºC under Ar for 8 h. After cooling to room temperature, the solvent was removed *in vacuo*. Purification by column chromatography (0→5→10→25% EtOAc/hexane) gave β-ketoamide **3c** (570 mg, 72%) as an yellow oil. ***Rotamer A*:** ^1^H NMR (400 MHz, COSY) δ 2.35 (q, J = 7.1 Hz, 2H, H-2’), 3.46 – 3.51 (m, 2H, H-1’), 3.70 (s, 2H, H-2), 4.02 (s, 2H, H-4), 4.66 (s, 2H, CH2Ph), 5.01 – 5.13 (m, 2H, H-4’), 5.61 – 5.87 (m, 1H, H-3’) 7.10 – 7.43 (m, 9H, Ph); ^13^C NMR (100 MHz, HSQC) δ 32.0 (C-2’), 39.6 (C-4), 46.1 (C-1’), 47.8 (CH2Ph), 48.6 (C-2), 51.7 (C-4), 117.0 (C-4’),127.2, 127.5, 128.0, 128.9, 129.1, 129.6, 132.2, 137.1 (Ph), 135.2 (C-3’), 167.1 (C-3), 201.0 (C-1). ***Rotamer B*:** ^1^H NMR (400 MHz, COSY) δ 2.24 (q, J = 7.1 Hz, 2H, H-2’), 3.20 – 3.25 (m, 2H, H-1’), 3.60 (s, 2H, H-2), 4.06 (s, 2H, H-4), 4.50 (s, 2H, CH2Ph), 5.01 – 5.13 (m, 2H, H-4’), 5.61 – 5.87 (m, 1H, H-3’) 7.10 – 7.43 (m, 9H, Ph); ^13^C NMR (100 MHz, HSQC) 32.8 (C-2’), 39.6 (C-4), 47.2 (C-1’), 47.9 (CH2Ph), 48.8 (C-2), 52.1 (C-4),118.0 (C-4’), 126.4, 127.3, 127.9, 128.8, 129.0, 129.6, 132.2, 136. 4 (Ph), 134.0 (C-3’), 166.9 (C-3), 200.9 (C-1). HRMS calcd for C_21_H_22_ClNO_2_ [M+H]^+^ 357.1310, found 357.1283.

***N*-benzyl-*N*-(but-3-en-1-yl)-4-(2-fluorophenyl)-3-oxobutanamide (3d).** To a mixture of *N*-benzylbut-3-en-1-amine (345 mg, 2.14 mmol), *tert*-butyl 4-(2-fluorophenyl)-3-oxobutanoate (810 mg, 3.21 mmol) in toluene (5 mL) was added 4-DMAP (79 mg, 0.642 mmol). The mixture was stirred at 80 ºC under Ar for 8 h. After cooling to room temperature, the solvent was removed *in vacuo*. Purification by column chromatography (0→5→10→25% EtOAc/hexane) gave β-ketoamide **3d** (620 mg, 85%) as a yellow oil. ***Rotamer A*:** ^1^H NMR (400 MHz, COSY) δ 2.25 (q, J = 7.1 Hz, 2H, H-2’), 3.23 (t, J = 12.0 Hz, 2H, H-1’), 3.59 (s, 2H, H-2), 3.92 (s, 2H, H-4), 4.49 (s, 2H, CH_2_Ph), 5.03 – 5.13 (m, 2H, H-4’), 5.61 – 5.87 (m, 1H, H-3’) 7.03 – 7.16 (m, 3H, Ar), 7.20 – 7.40 (m, 6H, Ar). ^13^C NMR (100 MHz, HSQC) δ 32.0 (C-2’), 43.3 (C-1’), 46.1 (C-2), 48.4 (CH_2_Ph), 51.5 (C-4), 117.0 (C-4’), 124.4, 126.4, 127.9, 128.8, 129.2, 129.3, 132.0, 136. 4 (Ar), 134.0 (C-3’), 162.8 (Ar-F), 167.1 (C-3), 200.9 (C-1). ***Rotamer B*:** ^1^H NMR (400 MHz, COSY) δ 2.35 (q, J = 7.1 Hz, 2H, H-2’), 3.48 (t, J = 8.0 Hz, 2H, H-1’), 3.70 (s, 2H, H-2), 3.96 (s, 2H, H-4), 4.66 (s, 2H, CH_2_Ph), 5.03 – 5.13 (m, 2H, H-4’), 5.61 – 5.87 (m, 1H, H-3’) 7.03 – 7.16 (m, 3H, Ar), 7.20 – 7.40 (m, 6H, Ar); ^13^C NMR (100 MHz, HSQC) δ 32.0 (C-2’), 43.3 (C-1’), 46.1 (C-2), 48.4 (CH_2_Ph), 51.5 (C-4), 117.0 (C-4’), 124.4, 126.4, 127.9, 128.8, 129.2, 129.3, 132.0, 136. 4 (Ar), 134.0 (C-3’), 162.8 (Ar-F), 167.1 (C-3), 200.9 (C-1). HRMS calcd for C_21_H_22_FNO_2_ [M+H]^+^ 340.1668, found 340.1663.

***N*-benzyl-*N*-(but-3-en-1-yl)-4-(2-nitrophenyl)-3-oxobutanamide (3e).** To a mixture of *N*-benzylbut-3-en-1-amine (192 mg, 1.19 mmol), *tert*-butyl 4-(2-nitrophenyl)-3-oxobutanoate (498 mg, 1.78 mmol) in toluene (3 mL) was added 4-DMAP (44 mg, 0.36 mmol). The mixture was stirred at 80 ºC under Ar for 8 h. After cooling to room temperature, the solvent was removed *in vacuo*. Purification by column chromatography (0→10→25→50% EtOAc/hexane) gave β-ketoamide **3e** (405 mg, 93%) as a yellow oil. ***Rotamer A*:** ^1^H NMR (400 MHz, COSY) δ 2.34 (dq, J = 14.7, 7.1 Hz, 2H, H-2’), 3.32 (t, J = 8.0 Hz, 2H, H-1’), 3.71 (s, 2H, H-2), 4.27 (s, 2H, H-4), 4.57 (s, 2H, CH_2_Ph), 5.02 – 5.13 (m, 2H, H-4’), 5.68 – 5.87 (m, 1H, H-3’) 7.19 – 7.64 (m, 9H, Ar). ^13^C NMR (100 MHz, HSQC) δ 32.0 (C-2’), 46.2 (C-1’), 47.3 (C-2), 48.5 (CH_2_Ph), 49.4 (C-4), 117.0 (C-4’), 125.3, 126.5, 127.5, 128.0, 128.7, 129.1, 133.9, 134.0, 136.4, 137.1 (Ar), 134.1 (C-3’), 148.6 (NO_2_-Ar) 166.9 (C-3), 199.9 (C-1); ***Rotamer B*:** ^1^H NMR (400 MHz, COSY) δ 2.34 (dq, J = 14.7, 7.1 Hz, 2H, H-2’), 3.50 (t, J = 12.0 Hz, 2H, H-1’), 3.84 (s, 2H, H-2), 4.30 (s, 2H, H-4), 4.68 (s, 2H, CH_2_Ph), 5.02 – 5.13 (m, 2H, H-4’), 5.68 – 5.87 (m, 1H, H-3’) 7.19 – 7.64 (m, 9H, Ar). ^13^C NMR (100 MHz, HSQC) δ 32.8 (C-2’), 46.2 (C-1’), 48.1 (C-2), 49.1 (CH_2_Ph), 52.1 (C-4), 118.1 (C-4’), 125.3, 126.5, 127.5, 128.0, 128.7, 129.1, 133.9, 134.0, 136.4, 137.1 (Ar), 134.1 (C-3’), 148.6 (NO_2_-Ar) 167.1 (C-3), 200.0 (C-1). HRMS calcd for C_21_H_22_N_2_O_4_ [M+H]^+^ 367.1613, found 367.1616.

***N*-benzyl-*N*-(but-3-en-1-yl)-3-oxo-4-(2-(trifluoromethyl)phenyl)butanamide (3f).** To a mixture of *N*-benzylbut-3-en-1-amine (204 mg, 1.27 mmol), *tert*-butyl 3-oxo-4-(2-(trifluoromethyl)phenyl)butanoate (574 mg, 1.91 mmol) in toluene (3 mL) was added 4-DMAP (46 mg, 0.38 mmol). The mixture was stirred at 80 ºC under Ar for 8 h. After cooling to room temperature, the solvent was removed *in vacuo*. Purification by column chromatography (0→5→10→25% EtOAc/hexane) gave β-ketoamide **3f** (412 mg, 84%) as a yellow oil. ***Rotamer A*:** ^1^H NMR (400 MHz, COSY) δ 2.24 (q, J = 8.7, 7.8 Hz, 2H, H-2’), 3.26 – 3.05 (m, 1H, H-1’), 3.52 – 3.43 (m, 1H, H-1’), 3.57 (s, 2H, H-2), 4.10 (dd, J = 14.2, 7.0 Hz, 2H, H-4), 4.48 (s, 2H, CH_2_Ph), 5.13 – 4.98 (m, 2H, H-4’), 5.63 (ddt, J = 25.2, 19.0, 9.8 Hz, 1H, H-3’), 7.14 (d, J = 7.0 Hz, 1H, Ar), 7.34 (dq, J = 24.9, 10.8, 9.0 Hz, 6H, Ar), 7.53 (t, J = 7.2 Hz, 1H, Ar), 7.66 (t, J = 8.5 Hz, 1H, Ar). ^13^C NMR (100 MHz, HSQC) δ 31.9 (C-2’), 46.2 (C-4), 48.4 (C-1’), 50.8 (CH_2_Ph), 52.0 (C-2), 117.1 (C-4’), 126.3, 127.5, 127.6, 127.9, 128.8, 129.1, 132.1, 132.2, 133.1, 133.2, 133.9 (Ar), 135.1 (C-3’), 136.2 (Ar), 167.0 (C-1), 200.9 (C-3). ***Rotamer B*:** ^1^H NMR (400 MHz, COSY) δ 2.34 (q, J = 7.0 Hz, 2H, H-2’), 3.26 – 3.05 (m, 1H, H-1’), 3.52 – 3.43 (m, 1H, H-1’), 3.68 (s, 2H, H-2), 4.10 (dd, J = 14.2, 7.0 Hz, 2H, H-4), 4.65 (s, 2H, CH_2_Ph), 5.13 – 4.98 (m, 2H, H-4’), 5.87 – 5.72 (m, 1H, H-3’), 7.14 (d, J = 7.0 Hz, 1H, Ar), 7.34 (dq, J = 24.9, 10.8, 9.0 Hz, 6H, Ar), 7.53 (t, J = 7.2 Hz, 1H, Ar), 7.66 (t, J = 8.5 Hz, 1H, Ar). ^13^C NMR (100 MHz, HSQC) δ 32.7 (C-2’), 47.2 (C-4), 48.7 (C-1’), 50.8 (CH_2_Ph), 52.0 (C-2), 118.1 (C-4’), 126.3, 127.5, 127.6, 127.9, 128.8, 129.1, 132.1, 132.2, 133.1, 133.2, 133.9 (Ar), 135.1 (C-3’), 136.9 (Ar), 167.3 (C-1), 200.9 (C-3). HRMS calcd for C_22_H_22_F_3_NO_2_ [M+H]^+^ 390.1636, found 390.1621.

**2-Benzyl-8-Hydroxy-7-phenyl-3,4-dihydroisoquinolin-1(*2H*)-one (5a).** This compound was prepared according to the above general procedure C using *N*-Benzyl-*N*-(but-3-en-1-yl)-3-oxo-4-phenylbutanamide (0.108 g, 0.336 mmol), Hoveyda-Grubbs 2^nd^ generation catalyst (0.011 g, 0.017 mmol), crotonaldehyde (0.118 g, 1.680 mmol), and Amberlyst 26 (0.284 g, 0.336 mmol). Purification by column chromatography (0→2.5→5→10% EtOAc/hexane) gave tetrahydroisoquinoline **9a** (0.062 g, 53%) as a brown oil. ^1^H NMR (400 MHz, COSY) δ 2.86 (t, *J* = 6.8 Hz, 2H, H-4), 3.43 (t, *J* = 6.8 Hz, 2H, H-3), 4.69 (s, 2H, Ph-CH_2_), 6.60 (d, *J* = 7.6 Hz, 1H, H-5), 7.32 – 7.15 (m, 7H, Ph), 7.35 (t, *J* = 7.6 Hz, 2H, Ph), 7.52 (d, *J* = 7.2 Hz, 2H, Ph), 13.18 (s, 1H, OH); ^13^C NMR (100 MHz, HSQC) δ 27.84 (C-4), 45.70 (C-3), 50.12 (CH_2_Ph), 117.28 (C-8a), 127.23, 127.85, 128.12, 128.26, 128.91, 129.38, 134.96 (C), 136.66 (C-4a), 137.96 (C), 159.11 (C-8), 168.83 (CO); HRMS calcd for C_22_H_19_NO_2_ [M+H]^+^ 330.1449, found 330.1440.

**2-benzyl-8-hydroxy-7-(*o*-tolyl)-3,4-dihydroisoquinolin-1(*2H*)-one (5b).** This compound was prepared according to the above general procedure C using *N*-benzyl-*N*-(but-3-en-1-yl)-3-oxo-4-(*o*-tolyl)butanamide (0.137 g, 0.408 mmol), Hoveyda-Grubbs 2^nd^ generation catalyst (0.013 g, 0.020 mmol), crotonaldehyde (0.143 g, 2.042 mmol), and Amberlyst 26 (0.116 g, 0.408 mmol). Purification by column chromatography (0→5→10→25% EtOAc/hexane) gave tetrahydroisoquinoline **9b** (0.088 g, 63%) as a brown oil. ^1^H NMR (400 MHz, COSY) δ 2.15 (s, 3H, CH_3_). 2.87 (t, *J* = 6.7 Hz, 2H, H-4), 3.44 (t, *J* = 6.7 Hz, 2H, H-3), 4.68 (s, 2H, Ph-CH_2_), 6.58 (d, *J* = 7.5 Hz, 1H, H-5), 7.34 – 7.01 (m, 10H), 12.90 (s, 1H, OH); ^13^C NMR (100 MHz, HSQC) δ 20.19 (CH_3_), 27.87 (C-4), 45.71 (C-3), 50.07 (CH_2_Ph), 116.94 (C-8a), 125.69, 127.74, 127.85, 128.15, 128.91, 129.94, 130.18, 135.31, 136.74 (C), 137.12 (C-4a), 137.88 (C), 159.05 (C-8), 168.80 (CO); HRMS calcd for C_23_H_21_NO_2_ [M+H]^+^ 344.1606, found 344.1592.

**2-benzyl-8-hydroxy-7-(2-chlorophenyl)-3,4-dihydroisoquinolin-1(*2H*)-one (5c).** This compound was prepared according to the above general procedure C using *N*-benzyl-*N*-(but-3-en-1-yl)-3-oxo-4-(2-chlorophenyl)butanamide (0.140 g, 0.395 mmol), Hoveyda-Grubbs 2^nd^ generation catalyst (0.012 g, 0.020 mmol), crotonaldehyde (0.138 g, 1.974 mmol), and Amberlyst 26 (0.334 g, 0.395 mmol). Purification by column chromatography (0→5→10→25% EtOAc/hexane) gave tetrahydroisoquinoline **9c** (0.070 g, 49%) as a brown oil. ^1^H NMR (400 MHz, COSY) δ 2.88 (t, *J* = 6.8 Hz, 2H, H-4), 3.44 (t, *J* = 6.8 Hz, 2H, H-3), 4.68 (s, 2H, Ph-CH_2_), 6.60 (d, *J* = 7.6 Hz, 1H, H-5), 7.43 – 7.38 (m, 1H, Ph), 7.31 – 7.15 (m, 9H), 12.99 (s, 1H, OH); ^13^C NMR (100 MHz, HSQC) δ 27.78 (C-4), 45.51 (C-3), 49.98 (CH_2_Ph), 116.71 (C-8a), 133.83, 131.90, 129.59, 128.88, 128.82, 128.08, 127.96, 127.77, 126.52, 135.43 (C), 136.57 (C-4a), 138.58 (C), 159.05 (C-8), 168.54 (CO HRMS calcd for C_22_H_18_ClNO_2_ [M+H]^+^ 365.0997, found 365.1010

**2-benzyl-7-(2-fluorophenyl)-8-hydroxy-3,4-dihydroisoquinolin-1(*2H*)-one (5d).** This compound was prepared according to the above general procedure C using *N*-benzyl-*N*-(but-3-en-1-yl)-3-oxo-4-(2-fluorophenyl)butanamide (0.155 g, 0.457 mmol), Hoveyda-Grubbs 2^nd^ generation catalyst (0.014 g, 0.023 mmol), crotonaldehyde (0.160 g, 2.283 mmol), and Amberlyst 26 (0.386 g, 0.457 mmol). Purification by column chromatography (0→5→10→25% EtOAc/hexane) gave tetrahydroisoquinoline **9d** (0.071 g, 45%) as a brown oil. ^1^H NMR (400 MHz, COSY) δ 2.88 (t, *J* = 6.7 Hz, 2H, H-4), 3.43 (t, *J* = 6.7 Hz, 2H, H-3), 4.69 (s, 2H, Ph-CH_2_), 6.60 (d, *J* = 7.6 Hz, 1H, H-5), 7.51 – 6.87 (m, 10H, Ph), 13.09 (s, 1H, OH); ^13^C NMR (100 MHz, HSQC) δ 27.88 (C-4), 45.64 (C-3), 50.12 (CH_2_Ph), 115.68 (C-8a), 115.90, 117.01, 123.17, 123.92, 123.96, 127.88, 128.17, 128.92, 129.31, 129.39, 132.05, 132.08, 135.74 (C), 136.66 (C-4a), 138.72 (C), 158.91 (C-8), 159.36, 161.37 (Ph-F), 168.67 (CO); HRMS calcd for C_22_H_18_FNO_2_ [M+H]+ 348.1355, found 348.1340.

**2-benzyl-8-hydroxy-7-(2-nitrophenyl)-3,4-dihydroisoquinolin-1(*2H*)-one (5e).** This compound was prepared according to the above general procedure C using *N*-benzyl-*N*-(but-3-en-1-yl)-3-oxo-4-(2-nitrophenyl)butanamide (0.096 g, 0.260 mmol), Hoveyda-Grubbs 2^nd^ generation catalyst (0.008 g, 0.013 mmol), crotonaldehyde (0.091 g, 1.303 mmol), and Amberlyst 26 (0.220 g, 0.260 mmol). Purification by column chromatography (0→10→25→50% EtOAc/hexane) gave tetrahydroisoquinoline **9e** (0.097 g, 52%) as a brown oil. ^1^H NMR (400 MHz, COSY) δ 2.88 (t, *J* = 6.7 Hz, 2H, H-4), 3.44 (t, *J* = 6.7 Hz, 2H, H-4), 4.66 (s, 2H, Ph-CH_2_), 6.65 (d, *J* = 7.6 Hz, 1H, H-5), 7.37 – 7.15 (m, 6H, Ph), 7.41 (q, *J* = 7.8 Hz, 2H, Ph), 7.57 (t, *J* = 7.6 Hz, 1H, Ph), 7.92 (d, *J* = 8.1 Hz, 1H, Ph), 13.05 (s, 1H, OH); ^13^C NMR (100 MHz, HSQC) δ 27.79 (C-4), 45.52 (C-3), 50.04 (CH_2_Ph), 117.56 (C-8a), 124.33 (C-6), 127.88, 128.05, 128.19, 128.43, 128.79, 128.92, 132.64, 132.90, 133.74 (C), 136.57 (C-4a), 139.01(C), 149.66 (C-NO_2_), 158.67 (C-8), 168.39 (CO); HRMS calcd for C_22_H_18_N_2_O_4_ [M+H]^+^ 375.1300, found 375.1293.

**2-benzyl-8-hydroxy-7-(2-(trifluoromethyl)phenyl)-3,4-dihydroisoquinolin-1(*2H*)-one (5f).** This compound was prepared according to the above general procedure C using *N*-benzyl-*N*-(but-3-en-1-yl)-3-oxo-4-(2-(trifluoromethyl)phenyl)butanamide (0.103 g, 0.264 mmol), Hoveyda-Grubbs 2^nd^ generation catalyst (0.008 g, 0.013 mmol), crotonaldehyde (0.093 g, 1.322 mmol), and Amberlyst 26 (0.223g, 0.264 mmol). Purification by column chromatography (0→5→10→25% EtOAc/hexane) gave tetrahydroisoquinoline **9f** (0.056 g, 53%) as a brown oil. ^1^H NMR (400 MHz, COSY) δ 2.89 (m, 2H, H-4), 3.45 (q, *J* = 6.0 Hz, 2H, H-3), 4.60 (d, *J* = 14.8 Hz, 1H, Ph-CH_2_), 4.75 (d, J = 14.8 Hz, 1H, Ph-CH_2_), 6.56 (d, *J* = 7.6 Hz, 1H, H-5), 7.12 (d, *J* = 7.5 Hz, 1H, Ph), 7.17 (s, 1H, Ph), 7.25 (dd, *J* = 16.1, 11.8 Hz, 5H, Ph), 7.39 (t, *J* = 7.6 Hz, 1H, Ph-3), 7.49 (t, *J* = 7.5 Hz, 1H, Ph-6), 7.68 (d, *J* = 7.8 Hz, 1H, Ph-4), 12.87 (s, 1H, OH); ^13^C NMR (100 MHz, HSQC) δ 27.86 (C-4), 45.62 (C-3), 50.07 (CH_2_Ph), 116.39 (C-8a), 127.79, 127.89, 128.19, 128.94, 131.40, 132.63, 135.09 (C), 136.70 (C-4a), 138.65 (C), 159.21 (C-8), 168.61 (CO); HRMS calcd for C_23_H_18_F_3_NO_2_ [M+H]^+^ 398.1323, found 398.1325.

**Scheme S1.** Robinson annulation reaction mechanism

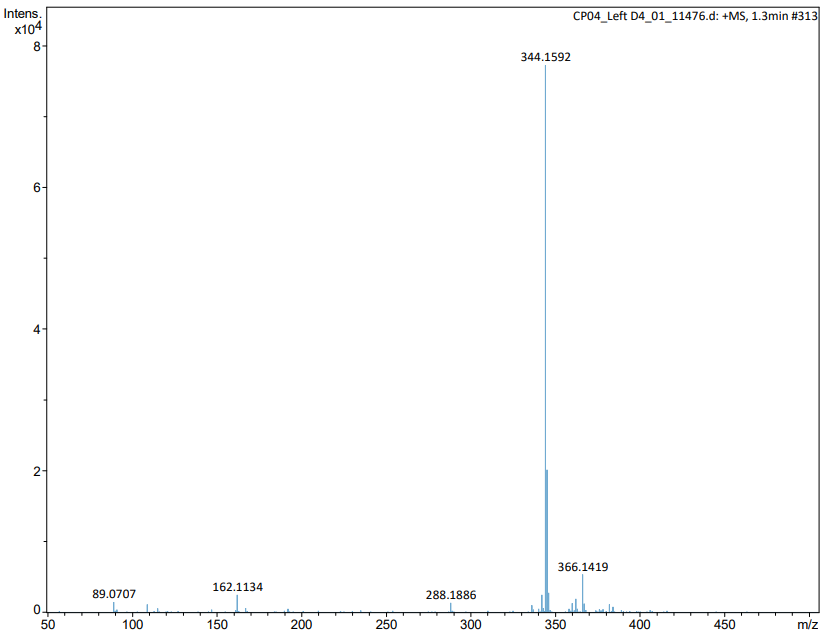


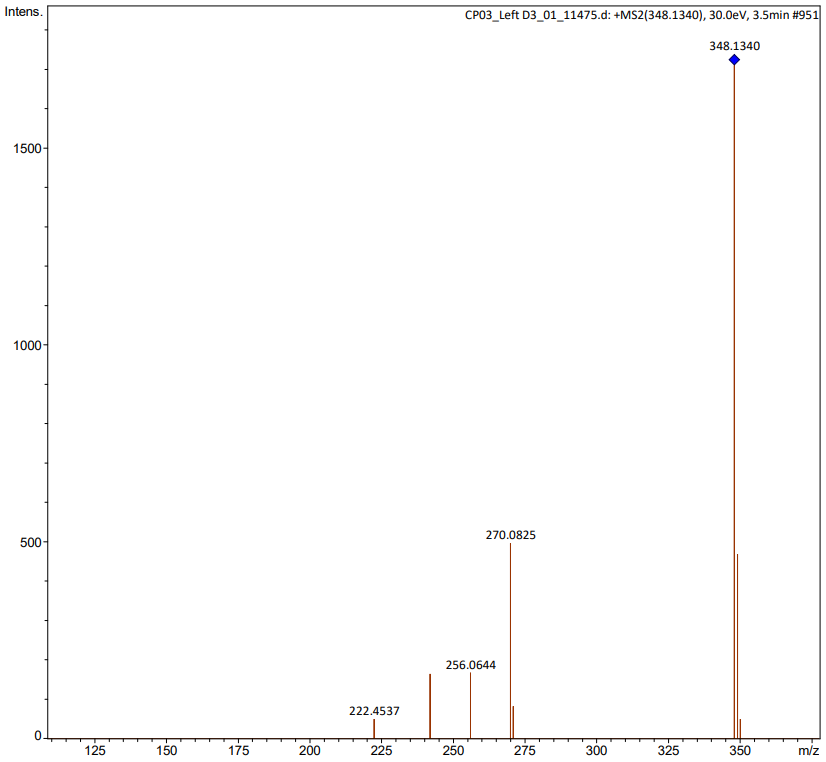


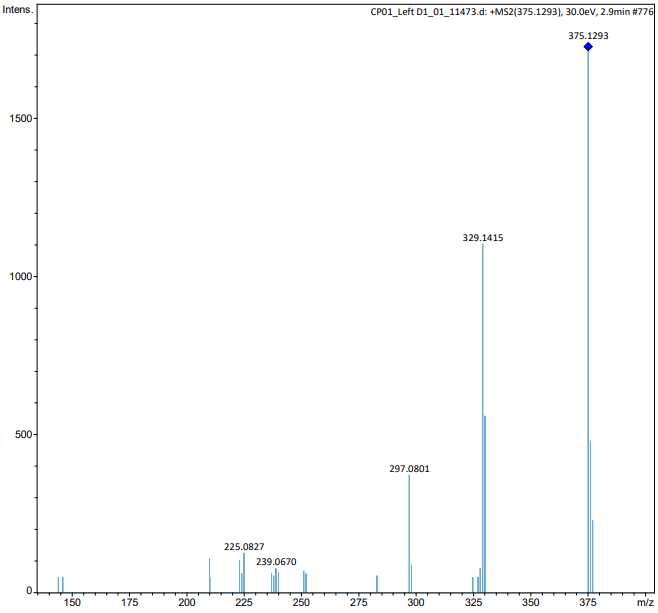


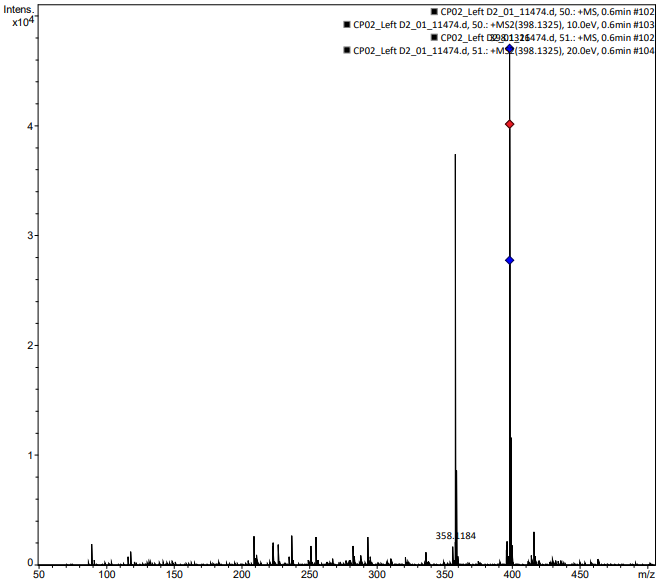


**References**

1 Martínez-Cifuentes, M. *et al.* Design of β-Keto Esters with Antibacterial Activity: Synthesis, In Vitro Evaluation, and Theoretical Assessment of Their Reactivity and Quorum-Sensing Inhibition Capacity. *Pharmaceuticals* **16** (2023). <https://doi.org/10.3390/ph16101339>

2 Bradshaw, B., Parra, C. & Bonjoch, J. Organocatalyzed Asymmetric Synthesis of Morphans. *Organic Letters* **15**, 2458-2461 (2013). <https://doi.org/10.1021/ol400926p>

3 Yin, Q., Wen, X., Chen, Y., Gong, X. & Hu, L. Phase-Transfer Catalyzed Asymmetric [4+1] Annulations for the Synthesis of Chiral 2,2-Disubstituted Tetrahydrothiophenes. *ORGANIC LETTERS* **23**, 7529-7534 (2021). <https://doi.org/10.1021/acs.orglett.1c02744>

4 Hofbauer, B. *et al.* Dual Inhibitor of &ITStaphylococcus aureus &ITVirulence and Biofilm Attenuates Expression of Major Toxins and Adhesins. *BIOCHEMISTRY* **57**, 1814-1820 (2018). <https://doi.org/10.1021/acs.biochem.7b01271>
